# Natural infection by *Procyrnea uncinipenis* (Nematoda, Habronematidae), a parasite from rheas, an autoctone bird from South America, in emus *Dromaius novaehollandiae*, a ratite from New Zealand

**DOI:** 10.1038/s41598-020-73754-1

**Published:** 2020-10-06

**Authors:** Nicole Brand Ederli, Samira Salim Mello Gallo, Francisco Carlos Rodrigues de Oliveira

**Affiliations:** 1 Instituto Do Noroeste Fluminense de Educação Superior (INFES), Universidade Federal Fluminense (UFF), Av. João Jasbick, s/n, Aeroporto, Santo Antônio de Pádua, RJ 28470-000 Brazil; 2 Laboratório de Sanidade Animal, Universidade Estadual Do Norte Fluminense Darcy Ribeiro (UENF), Campos dos Goytacazes, RJ 28013-602 Brazil

**Keywords:** Parasitology, Parasite biology, Microbiology

## Abstract

The present study reports a natural infection of emus, *Dromaius novaehollandiae*, by the nematode *Procyrnea uncinipenis*. Five adult emus from a scientific breeding farm at North Fluminense State University located in the city of Campos dos Goytacazes, Rio de Janeiro state, Brazil were necropsied, and their gastrointestinal tract were collected and examined for the presence of parasites from October 2013 to November 2015. Two of the five (40%) emus necropsied were infected with nematodes, and a portion of the nematodes were processed for light microscopy. In addition, two other nematodes (a male and a female) were prepared for scanning electron microscopy. In a female bird, one nematode was collected in the proventriculus and two nematodes in the gizzard and in the male bird four nematodes were collected in the gizzard. The morphological and morphometric analyzes allowed to identify the nematodes as *P. uncinipenis*, this being the first report of an infection by *P. uncinipenis* in emus. Therefore, we infer that these emus were naturally infected by nematodes that were considered specific to rheas.

## Introduction

There are two species of rheas in South America, the greater rhea, *Rhea americana* Linnaeus, 1758, and the lesser rhea, *Rhea pennatta* (d'Orbigny 1834). Of these, *R. americana* has been of increased interest for farming since the 1990s, receiving attention in South America, North America and Europe^1^. This species is composed of five subspecies distributed in South America: *R. americana americana* (northeastern to southeastern Brazil), *R. americana intermedia* (southeastern Brazil and Uruguay), *R. americana nobilis* (eastern Paraguay), *R. americana araneipes* (western Paraguay, eastern Bolivia, and southwestern Brazil) and *R. americana albescens* (northeastern and eastern Argentina)^2^. These birds are found in open and stream areas^3^, and the presence of parasitic infections in these species is common, particularly those caused by the nematodes *Procyrnea uncinipenis* (Molin, 1860) and *Deletrocephalus dimidiatus* Diesing, 1851, *Deletrocephalus cesarpintoi* Vaz, 1936 and *Paradeletrocephalus minor* (Molin, 1861)^4,5^, both of which are considered host-specific.

The emu, *Dromaius novaehollandiae* (Latham 1790), is widely distributed throughout the Oceania continent and is one of the most characteristic components of the modern Australian avifauna^6^. There are three living subspecies: *D. novaehollandiae novaehollandiae* (central and south Queensland, Victoria and Southern Australia); *D. novaehollandiae woodwardi* (Northwestern and Western Australia and northern territory), and *D. novaehollandiae rothschildi* (Southwestern Australia)^7^. In Brazil, these birds have been farmed for commercial and ornamental purposes. Studies regarding parasitic infection in these birds are scarce, with no reports of species that infect other ratites.

*Procyrnea uncinipenis* is a nematode that infect the gizzard and proventriculum of rheas, *Rhea americana*, a native bird from South America. These nematodes have been reported in rheas from Brazil^4,8–11^ and Argentina^12^. Another species, *Procyrnea choique*^13^, was described from another species of rhea, *R. pennatta*, from Argentina.

The present study reports a natural infection of emus, *D. novaehollandiae*, by the nematode *P. uncinipenis*.

## Material and methods

Five adult emus (3 males and 2 females) from a scientific breeding farm regulated by the governmental agency IBAMA under number 18981-2 and approved by Ethics Committee at the Universidade Estadual do Norte Fluminense (North Fluminense State University) located in the city of Campos dos Goytacazes, State of Rio de Janeiro, Brazil were necropsied after natural death, and the gastrointestinal tract were collected and examined for the presence of parasites from October 2013 to November 2015. All applicable institutional, national and international guidelines for the care and use of animals were followed. These birds live in proximity to *R. americana* from the same breeding*.* The contents of the proventriculum and gizzard were passed through a sieve with a 75 µm mesh, and the mucosa was observed under a stereomicroscope (Opton TIM-2T, China). The koilin layer was removed and observed for the presence of nematodes. The nematodes that were found were washed in a saline solution (0.09% NaCl). A portion of the nematodes were processed for light microscopy, and two (a male and a female) were prepared for scanning electron microscopy.

### Optical microscopy

The nematodes were fixed in A.F.A. (70° GL ethanol, 93%; formaldehyde, 5%; glacial acetic acid, 2%) at 70 °C, for 48 h, transferred to a solution containing 70% ethanol and 5% glycerin, cleared and mounted on slides with lactophenol (one part distillated water, two parts glycerin, one part lactic acid, one part phenic acid) and observed under a light microscope.

Measurements were performed to the nearest micron (mean ± S.D. (range)) and were conducted on three mature adult specimens and 10 embryonated eggs in utero. Measurements were conducted with an Axioplan Zeiss light microscope (Carl Zeiss, Germany) equipped with a Canon Power-Shot A640 digital camera (Canon, China) and Zeiss AxionVision Sample Images Software (Carl Zeiss, Germany) for image analysis. Specimens deposited in the Harold W. Manter Parasite Collection at the University of Nebraska-Lincoln (UNL/USA), registration number HWML 67092, were examined for comparative purposes. Representative specimens were deposited in the Helminthological Collection of the Oswaldo Cruz Institute (Rio de Janeiro, Brazil) under the registration number CHIOC 38941.

### Scanning electron microscopy

The nematodes were fixed for 2 h in 2.5% glutaraldehyde, 4% freshly prepared paraformaldehyde, and 5 mM calcium chloride in 0.1 M cacodylate buffer, pH 7.2. The nematodes were postfixed in 2% osmium tetroxide in 0.1 M cacodylate buffer. The samples were dehydrated in an acetone series, critical-point dried with CO_2_, sputter-coated with gold and examined under a Zeiss 962 scanning electron microscope (SEM) operating at 15 kV.

### Ethical approval

All applicable institutional, national and international guidelines for the care and use of animals were followed.

## Results

Two of the five (40%) analyzed emus (one male and one female) were infected with nematodes. In one bird (female), one nematode was collected from the proventriculus and two from the gizzard, under the koilin layer. In another bird (male), four nematodes were collected from under the gizzard koilin layer. No gross pathology was observed.

**Figure 1:**
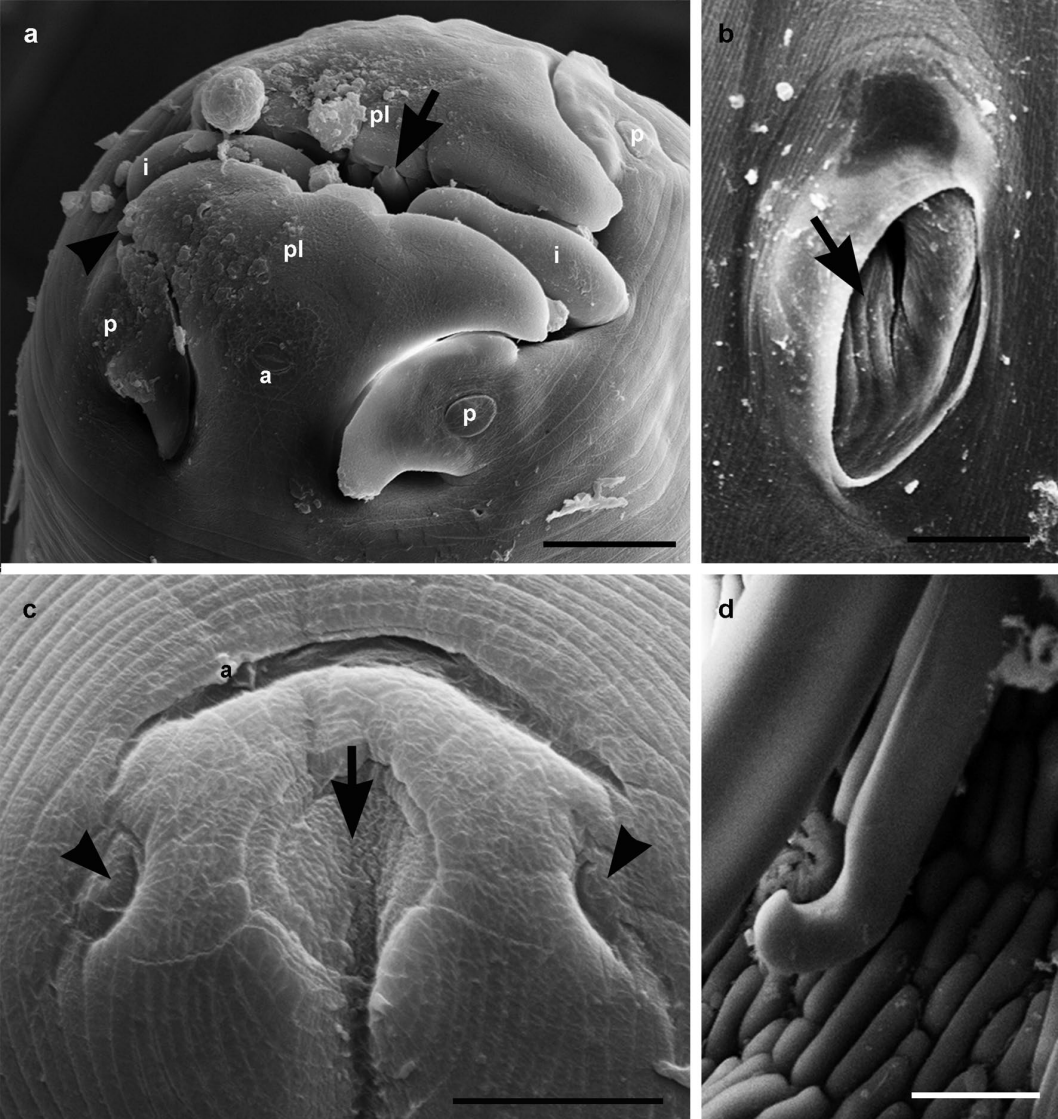
Scanning electron microscopy of *Procyrnea uncinipenis* (Nematoda, Habronematidae) from *Dromaius novahollandiae* (Aves, Casuariidae). (**a**) Anterior end showing lateral pseudolips (pl), interlabia (i), cephalic papillae (p), amphidia (a), pseudolip denticles (arrow) and lateral lip denticles (head arrow); (**b**) right spicule, distal end (arrow); (**c**) female tip tail showing two lateral phasmids (head arrow), a circular structure (arrow) and the anus (a); (**d**) vulva opening (arrow). Bars: A–B: 25 µm, C: 15 µm, D: 50 µm.

The morphological and morphometrical analyses identified the nematodes as *P. uncinipenis*. The nematodes were large and whitish in vivo and had two lateral lips with denticles and two interlabia, four labial papillae and two amphids (Fig. 1a). The esophagus was divided into an anterior muscular portion and a posterior glandular portion. Only one female specimen was measured, which had a total body length approximately 21,724 by 734 wide. Buccal cavity 55 long by 38 wide. Muscular esophagus 357 long by 93 wide, and glandular esophagus 4874 long by 306 wide. Distances from nerve ring, excretory pore and cervical papillae to anterior end were approximately 289, 1368 and 1401, respectively. Anus and vulva with a transverse slit (Fig. 1b,c) opening at 236 and 1484 from posterior end, respectively. Female posterior end with two lateral phasmids and a circular structure at the tip tail (Fig. 1c). Eggs (n = 10) 26 ± 1.31 long, ranging from 24 to 28, by 43 ± 2.36 wide, ranging from 40 to 48.

**Figure 2:**
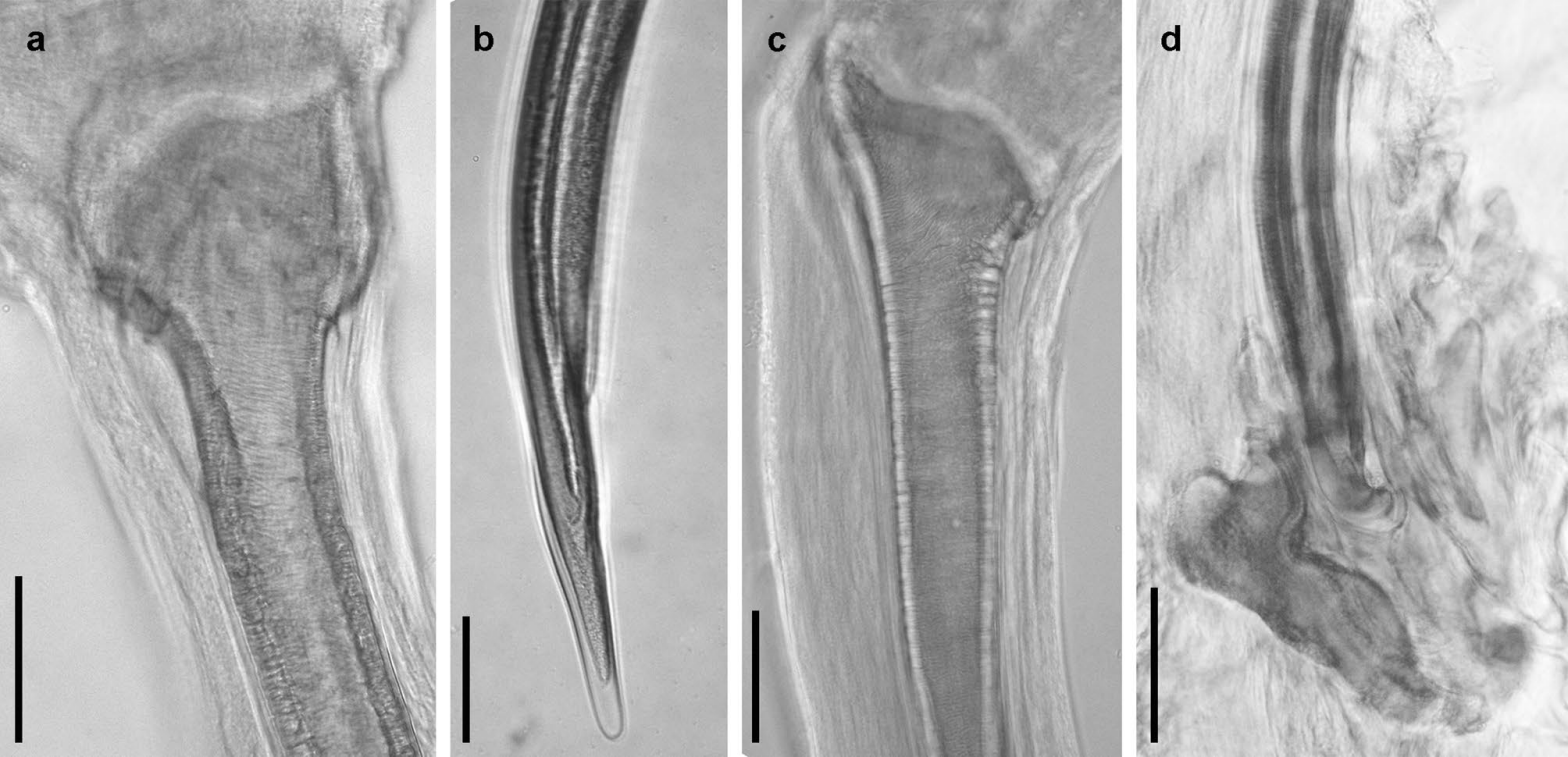
Light microscopy of the spicules of *Procyrnea uncinipenis* (Nematoda, Habronematidae) from *Dromaius novahollandiae* (Aves, Casuariidae). (**a**) Proximal end of the left spicule; (**b**) distal end of the left spicule; (**c**) proximal end of the tight spicule; (**d**) distal end of the right spicule. Bars: 50 µm.

Two males were measured, and the total body length ranged from 14,855 to 20,861 long by 624 to 625 wide, buccal cavity 47–65 long by 42–60 wide, muscular esophagus 313–516 long by 104–108 wide, glandular esophagus 3305–3442 long by 264–266 wide, nerve ring at 422–432 from anterior end. Excretory pore was not observed in the specimens. Spicules unequal in size and shape, with a proportion of approximately 1:4. Left spicule long and thin, measuring 3224 long, with a more robust spicule head (Fig. 2a). Distal tip ended sharply (Fig. 2b). Spicule head shorter (Fig. 2c). Right spicule short and thick, measuring 741 long, with a curved distal end, similar to a hook, and a dilation at the tip (Figs. 1d, 2d). Gubernaculum well chitinized, “v” shaped (Fig. 2d), 145 long.

## Discussion

The morphology, observed by light and scanning electron microscopy, and the morphometry of the nematodes collected from *D. novaehollandiae* are similar to those of the nematode species that infect *R. americana* in South America, *P. uncinipenis*, which shows that this exotic bird can host this parasite from rheas, a native bird of the continent. This is the first report of an infection by *P. uncinipenis* in emus. Other studies that evaluated this species of parasites in emus have not reported the presence of *P. uncinipenis*^14,15^. There are reports of infection by other nematode species, including *Chandlerella quiscali*^16^, *Baylisascaris* spp.^17^, *Cyathostoma variegatum*^18^, *Dromaestrongylus bicuspis*, *Trichostrongylus tenuis*, and *Syngamus trachea*^19^.

This report of *P. uncinipenis*, a parasite from *R. americana*, in emus bred in captivity in Brazil together with rheas shows that these birds developed an adaptation to this nematode parasite, which was considered to be host-specific. In rheas, there can be a high intensity infection, with more than 400 nematodes infecting the proventriculus and gizzard, causing widespread necrosis accompanied by a hemorrhagic appearance. The nematodes deeply penetrate the gizzard glands^4^. Although the emus had few specimens of *P. uncinipenis* in the gizzard (n = 4), infections with a high parasite load can occur and lead to the death of the birds.

The female and male specimens of *P. uncinipenis* that infected the emus in the present study are smaller than those from *R. americana* and larger than *P. choique* from *R. pennata* (Tables 1 and 2). However, the morphology observed by light and scanning electron microscopy is similar to *P. uncinipenis*, a parasite from *R. americana*. These differences in nematode size are probably due to early infection in emus. The low prevalence (2 of 5 analyzed birds) and low parasite load (one bird with 3 nematodes and another with 4 nematodes) shows that this ratite species, *D. novaehollandiae*, originally from New Zealand, become naturally infected by this nematode species from another ratite from South America, *R. americana*. Thus, farmers and zoological gardens from Oceania should pay attention to infection with *R. americana* in their flock by using effective sanitary control measures. The intention should be to avoid the introduction of this parasite, which has the capacity to infect emus. The consequences of this parasite in other birds are unknown. In rheas, this parasitosis can lead to death^4^. *Procyrnea uncinipenis*, as a Spirurid, has an arthropod as intermediate host, which is unknown. So, it is not known if this parasite can complete its life cycle, if introduced in Oceania.

**Table 1:** Measurements, in microns, of male specimens of *Procyrnea uncinipenis*, a parasite of rhea.

Character	*P. uncinipenis* present study (n = 2)	*P. uncinipenis*^20^	*P. uncinipenis*^9^	*P. uncinipenis*^8,b^	*P. waltoni*^21,c^	*P. choique*^13,d^
Body total length	14,855–20,861	22,546–30,980	17,750–21,770	25,000–28,000	20,000	8250–9850
Body width	624–625	659–827	600–740	600–700	700	350–400
Buccal cavity deep	47–65	46–68	55–63	62	120–130	30–40
Buccal cavity wide	42–60	24–40	67–84	37	–	25–30
Muscular esophagus length	313–516	351–455	300–360	400–420	425–450	200–300
Muscular esophagus width	104–108	74–108	70–83		–	40
Glandular esophagus length	3305–3442	2685–3568	2660–3650	3220–3400	2600–2900	2640–3560
Glandular esophagus width	264–266	164–249	170–230		–	80–120
Nerve ring^a^	422–432	282–490	330–390	420	360–400	120–200
Excretory pore^a^	–	529–658	500–610		–	250–290
Cervical papillae^a^	–	361–411	290–340	–	–	140–180
Right spicule length	741 (n = 1)	740–982	660–800	700–720	300–420	300–360
Left spicule length	3224 (n = 1)	3206–3774	3000–3700	3050–3170	2400–2650	970–1050
Right spicule:left spicule	1:4	1:4	1:4	1:4	1:8	~ 1:4
Gubernaculum length	145	102–169	110–140	100	–	60–80

^a^Measured from the anterior end.

^b^Described as *Sicarius nobregai* (synonym of *P. uncinipenis*).

^c^Described as *S. uncinipenis* (synonym of *P. waltoni*).

^d^Parasite of *Rhea pennata.*

**Table 2:** Measurements, in microns, of female specimens of *Procyrnea uncinipenis,* a parasite of rhea.

Characters	*P. uncinipenis* present study (n = 1)	*P. uncinipenis*^20^	*P. uncinipenis*^9^	*P. uncinipenis*^8,c^	*P. waltoni*^21,d^	*P. choique*^13,e^
Body total length	21,724	35,174–43,319	26,460–33,160	33,000–36,000	25,000	13,400–17,800
Body width	734	887–1055	670–800	700–900	750	400–600
Buccal capsule deep	55	44–80	52–70	62	120–140	45–50
Buccal capsule width	38	30–48	78–96	37	–	30–40
Muscular esophagus length	357	393–737	320–410	400–420	390–410	340–460
Muscular esophagus width	93	78–143	91–104	–	–	40–55
Glandular esophagus length	4874	3350–4684	3490–3980	3220–3400	3250–3550	1870–5300
Glandular esophagus width	306	195–360	220–230	–	–	70–80
Nerve ring^a^	289	387–786	390–400	420	360–400	250–330
Excretory pore^a^	1368	652–804	530–650	–	–	350–550
Cervical papillae^a^	1401	319–952	340–400	–	Not observed	170–230
Vulva^b^	1484	13,596–17,766	11,050–12,060	16,300–16,500	6600–7100	6200–7100
Tail	236	180–287	220–250	260	260	80–100
Eggs length	40–48	35–51	46–50	47	45–50	50–55
Eggs width	24–28	21–32	25	25	24–26	30–35

^a^Measured from the anterior end.

^b^Measured from the posterior end.

^c^Described as *Sicarius nobregai* (synonym of *P. uncinipenis*).

^d^Described as *S. uncinipenis* (synonym of *P. waltoni*).

^e^Parasite of *Rhea pennata.*

After analyzing the nematode specimens collected from the gizzard of emus, a bird from Oceania that were introduced in Brazil for commercial and ornamental purposes, along with rheas, a native bird from South America, the present study can infer that the emus were naturally infected by nematodes that were considered specific to rheas.
